# Confidential Reports and Their Abuses

**Published:** 1913-12-13

**Authors:** 


*To the Editor of* The Hospital.

### Confidential Reports and their Abuses.

*To the Editor of* The Hospital.

Sir,—You have had occasion at times to comment on the official procedure in dealing with matters of interior economy of hospital administration, as an instance of which I may refer to the article on "Resident Medical Officers and Ward Sisters," in your issue of July 12 last, in which, quite apart from the merits of the case, it is obvious that the procedure of the Committee opened wide the door for a sense of injustice, instead of rendering such a thing impossible.

Your article affords ground for the consideration of another matter which closely concerns the life of a nurse under training, and largely makes for contentment or otherwise. I refer to the subject of the "Confidential Reports," and cognate to it is the subject of verbal reports, on which a nurse may be summoned before such body of the administration as may have to deal with it. Naturally, such laxity of administration opens the door to still less desirable features, and I have known a nurse summoned before a Committee without having any idea of the reason for which she was summoned, and a perfectly valid sense of gross and real injustice thus induced. One would not like to think that such a want of system prevailed in many hospitals in the country, but the prevention of such an injustice, or even a sense thereof, could so easily and obviously be carried out by a rule that any nurse whom it is proposed to summon before a Committee should be provided beforehand with a statement in writing of the cause, and that she should have a chance of defending herself or pleading mitigation.

Now for the confidential reports. It may not be amiss, first of all, briefly to recapitulate the main objects of confidential reports, in any department of employment. The main objects may be defined as twofold: (1) To bring to notice the varied abilities and qualifications of those employed, with a view to their selection, if necessary, for promotion or for some particular work for which they are peculiarly qualified; (2) to enable employers concerned to weed out those who are incompetent or useless. If the reports began and ended there, with an unequivocal and irreproachable statement of facts supported by evidence, they would make for nothing but good, and good for every one, employers and employed alike. But such is not the case. There is an element which will creep in, more or less according to the facilities afforded it, and means should be adopted to reduce such a possibility to an irreducible minimum. Human factors, such as like and dislike, partiality and impartiality, bias and prejudice, cannot be eliminated, and as a matter of simple justice those reported on should be protected from being "stabbed in the back" as much as possible and every one given, on broad principles of justice, the world-wide right of defence. As regards the procedure adopted in the case of a nurse being badly reported on, I can only speak of those hospitals with which I am familiar, so I do not know what variations of procedure there may be, though I quite expect it is practically the same all over the country. The nurse is told that So-and-So has made a bad report, and five times out of six there may be nothing to be said or done, except for the nurse to improve or amend her ways. But the sixth time the nurse smarts under a sense of injustice, and she may have such a defence to offer as would cancel the adverse report. Now, it is not enough for the nurse to be told of an adverse report; she should read it herself in detail. They are two very different things. Indeed, it would be very much better and fairer if she were given a copy of the report, so that she might prepare a defence if she thought fit; a defence she would in all probability not be able to offer in the worry of having a "wigging." (I may say here, in parenthesis, that I can give you chapter and verse in support of every word of this letter.) In the event of the nurse having a defence to offer, facility should be given her for submitting it, but such is not always the case, rather the contrary. Granted that the defence may be bad, she should be afforded facilities for making it, if only in common justice.

Not only should adverse reports be read in detail by the person reported on, but the person making the report should know that it will be so read, and that the person reported on will be given facilities for making a defence, and therein lies the means of eliminating as far as possible those factors referred to above; and if any of those factors should have crept in, and the defence has proved good, again in common fairness the person reported on should be told the adverse report does not hold good. There is one other point which may be referred to, and that is, it is a distinct and valuable encouragement to zeal and keenness for those well reported on to be told of it also, and I have not yet been given a reason why it should not be done.

It would be a good thing, I think, if standard rules could be drawn up generally applicable to all hospitals and incorporated in the Rules and Regulations for the Nursing Staff, so that they could be read and understood by every member of the staff, senior and junior. At present a perfectly unnecessary amount of heart-burning, worry, uncertainty and anxiety is dependent on the existing system of confidential reports, which could so easily be obviated—as I believe your readers will agree.—Yours, etc.,

Hospital Officer.

